# Effect of Nedocromil Sodium on Aspecific Bronchial Hyper-Reactivity in Asthmatic Children

**DOI:** 10.1155/S096293519400075X

**Published:** 1994

**Authors:** A. Fiocchi, P. Signoroni, P. Bruni, M. Galeone, E. DeCet, S. Bogacki

**Affiliations:** 5th Paediatric Department University of Milan Via Rossini, 2 Milan 20062 Italy

## Abstract

The purpose of this study was to evaluate whether nedocromil sodium benefits urban asthmatic children showing seasonal bronchial hyper-reactivity to ultrasonic nebulization of distilled water (UNDW). A prospective, randomized, placebo-controlled, parallel-group, double-blind study was carried out at the outpatient pulmonology service at a tertiary-care teaching hospital. Twelve children living in Milan, who were 7–17 years of age, who were SPT and RAST-negative to perennial allergens, who were suffering from episodic asthma, and showing seasonal bronchial hyper-reactivity to UNDW during winter, participated in this study. All the children received either placebo or nedocromil sodium, 4 mg every 6 h for 6 weeks. Spirometry and UNDW challenge were done at the following times: day−7; day 0; day 1; day 7; day 14; day 28; day 42. No differences were found in the basal spirometric parameters, which were normal in both nedocromil and placebo groups. Bronchial reactivity to UNDW was found to be significantly decreased in the group treated with nedocromtl starting from day 7. It is therefore concluded that nedocromil sodium can reverse bronchial hyper-reactivity caused by seasonal factors such as cold, viral infections and atmospheric pollutants in children suffering from asthma.


Mediators of Inflammation 3, S43–S47 (1994)

MOIA15

### Effect of nedocromil sodium on aspecific bronchial hyper-reactivity in asthmatic children

A. Fiocchi,CA P. Signoroni, P. Bruni, M. Galeone, E. DeCet and S. Bogacki

5th Paediatric Department, University of Milan, Via Rossini, 2, 20062 Milan, Italy

CA Corresponding Author

**The purpose of this study was to evaluate whether nedocromil sodium benefits urban asthmatic children showing seasonal bronchial hyper-reactivity to ultrasonic nebulization of distilled water (UNDW). A prospective, randomized, placebo-controlled, parallel-group, double-blind study was carried out at the outpatient pulmonology service at a tertiary-care teaching hospital. Twelve children living in Milan, who were 7–17 years of age, who were SPT and RAST-negative to perennial allergens, who were suffering from episodic asthma, and showing seasonal bronchial hyper-reactivity to UNDW during winter, participated in this study. All the children received either placebo or nedocromil sodium, 4 mg every 6 h for 6 weeks. Spirometry and UNDW challenge were done at the following times: day –7; day 0; day 1; day 7; day 14; day 28; day 42. No differences were found in the basal spirometric parameters, which were normal in both nedocromil and placebo groups. Bronchial reactivity to UNDW was found to be significantly decreased in the group treated with nedocromil starting from day 7. It is therefore concluded that nedocromil sodium can reverse bronchial hyper-reactivity caused by seasonal factors such as cold, viral infections and atmospheric pollutants in children suffering from asthma.**

**Key words:** Asthma, Bronchial hyper-reactivity, Children, Nedocromil, Ultrasonic nebulized distilled water challenge, UNDW

### Introduction

Bronchial hyper-reactivity (i.e. exaggerated bronchoconstrictive response to various stimuli) is a characteristic of asthma both in children and adults.[1,2] Bronchial hyper-reactivity is, in part, genetically determined and, in part, a consequence of airway inflammation,[3–6] and it is closely related to the severity of the disease.[7] Studies performed on animal models show that exposure to such environmental pollutants as $SO_4$ and ozone may induce bronchial hyper-reactivity,[8] and it has been suggested that climate-therapy may attenuate these effects.[9,10] In children living in urban areas, exposure to pollution has been associated with an increase in the number of cases of secretive chronic bronchitis and to a reduction in respiratory function,[11] as well as to an increase in asthma[12] and to a higher risk of allergic sensitization.[13]

Anti-inflammatory drugs, such as inhaled corticosteroids and cromones, are widely used to reverse bronchial hyper-reactivity.[14–20] The aim of this study was to evaluate the protective effect of nedocromil sodium on bronchial hyper-reactivity in a group of non-atopic asthmatic children who were showing seasonal bronchoreactivity, and who were living in town.

### Patients and Methods

From 1 November to 31 December 1990, children attending the Allergology and Pneumology Out-Patients Clinic of the 5th Pediatric Department of the University of Milan were recruited according to the following criteria: age 6–18 years; suffering from bronchial asthma; living and attending school in Milan; skin tests and RAST negative for perennial allergens (house dusts, dermatophagoides, animal dander); seasonal bronchoreactivity, assessed as: (1) positive aspecific challenge to ultrasonic mist performed between 1 October 1989 and 31 March 1990 (time A); (2) negative challenge performed between 1 August and 30 September 1990 (time B); and (3) positive challenge performed upon recruitment (time C).

Exclusion criteria were: non-response to challenge with ultrasonic nebulized distilled water (UNDW) at time A or C; response to challenge with UNDW at time B; positive skin tests for perennial inhalant allergens; airway infection within 30 days of the tests; intake of β-2 agonists, theophylline, cromones, corticosteroids, antihistamines and ketotyphen, 6 h before testing. The relevant features of the patients of the two groups are shown in Table 1.

Patients inhaled UNDW, 5 ml/min per 5 min,

© 1994 Rapid Communications of Oxford Ltd

**Table 1.** Relevant features of the patients of the two groups

| Group N | | | | | Group P | | | | |
|---|---|---|---|---|---|---|---|---|---|
| Patient | Sex | Age (yrs) | Weight (kg) | Height (cm) | Patient | Sex | Age (yrs) | Weight (kg) | Height (cm) |
| 1 | M | 7.5 | 22 | 126 | 2 | M | 16.2 | 67 | 181 |
| 3 | M | 12.4 | 45 | 152 | 6 | M | 15.7 | 68 | 185 |
| 4 | M | 11.7 | 40 | 156 | 7 | M | 7.1 | 27 | 125 |
| 5 | F | 9.1 | 30 | 132 | 8 | M | 14.1 | 54 | 173 |
| 9 | F | 8.1 | 32 | 134 | 11 | M | 12.3 | 50 | 159 |
| 10 | F | 13.2 | 53 | 159 | | | | | |
| 12 | M | 11.7 | 37 | 149 | | | | | |

delivered by a Fujikogaru ultrasonic nebulizer (Osaka, Japan) that produces 5 μm particles of water. UNDW was administered according to Allegra and Bianco.[21] A test was judged positive when after challenge FEV1 (forced expiratory volume in 1 s) dropped 20% or more, and/or MMEF (maximal mid-expiratory flow) dropped 35% or more. Spirometric evaluations and flow/volume curve recordings were always performed at the same time (14.00–16.00 h), 6 h after the last administration of nedocromil, using a Spirostar FG 90 spirometer (Werner-Gut AG, Bern, CH), equipped with a n.2 Fleisch pneumotachograph; reference parameters were according to Zapletal.[22] Before challenge, a basal spirometric examination was performed, and measurements were taken at 1, 2, 5, 10 and 15 min after the challenge. Fourteen children fulfilling the above-listed criteria were included in this trial. The subjects were nine males and five females, aged 7.33–16.92 years (mean age: 10.12 ± 3.52 years; median age: 11.40 years). After informed consent was obtained, children were randomly assigned to treatment with nedocromil sodium, 2 puffs four times per day for 42 days (group N; seven subjects), or to the placebo group (group P).

In case of asthmatic episodes, salbutamol could be taken (Ventolin MDI, Glaxo, Verona, Italy), at a dose of 1 puff, corresponding to 200 μg, up to four times daily.

Cromones, corticosteroids and antihistamines were not allowed throughout the study period. Aspecific challenge by inhalation of UNDW was performed at the following times: –7 days (time–7); day 0 (time 0); day 1 (time 1); day 7 (time 7); day 14 (time 14); day 28 (time 28); day 42 (time 42). Data are expressed in percentages with respect to expected values, according to the formula:

$$\frac{\text{measured value}}{\text{expected value}} \times 100$$

The results of the aspecific bronchoreactivity test are expressed in percentages with respect to baseline as follows:

$$\frac{\text{baseline} - \text{post-challenge value}}{\text{baseline}} \times 100$$

Evaluation was performed with Mann–Whitney's non parametric tests, and Wilcoxon's rank-sum test.

### Results

Two girls of group P dropped out of the study; one because of an airway infection, probably caused by a virus, and the other because of an asthma attack that was treated with systemic steroid. The data of these two subjects were not included in the final evaluation. Therefore, 12 children completed the study: nine male and three female, aged 7.33–16.92 years (mean age: 11.86 ± 2.04 years; median age: 12.20 years). Seven subjects (four male and three female; aged 7.42–13.42 years; mean age: 10.81 ± 2.16 years; median age: 11.33 years) belonged to group N, and five males constituted group P (age 7.33–16.92 years; mean age: 13.33 ± 3.71 years; median age: 14.42 years). No significant age-related difference was found between the two groups ($f$ = 2.23; $t$ = 1.49; $p$ = N.S.).

*Basal spirometric parameters (baseline/expected):* As shown in Fig. 1, there was no difference at any time for VC (vital capacity; baseline/expected), PEF (peak expiratory flow), FEV1 and FEF25-75 (forced expiratory flow between 25 and 75% of vital capacity) between the treated and placebo groups. The data show a parallel trend which was fairly constant in time; therefore, nedocromil sodium treatment did not affect the parameters measured.

*Bronchoreactivoty to challenge with UNDW:* Fig 2 shows the median percentage variations (UNDW/baseline) of vital capacity, FEV1, PEF, and FEF25-75 in the treated and in the placebo group. The change in vital capacity after UNDW was substantially less in the treated group, even before therapy ($t$ = –7), but the difference became significant during treatment (Table 2). At day –7, FEV1 changes after UNDW were similar in the two groups. Differences were observed at day 7: bronchoreactivity in the placebo group remained stable, and decreased significantly in the treated group (Table 3).

The profile of PEF after UNDW was almost parallel in the two groups; only at day 42 was there a

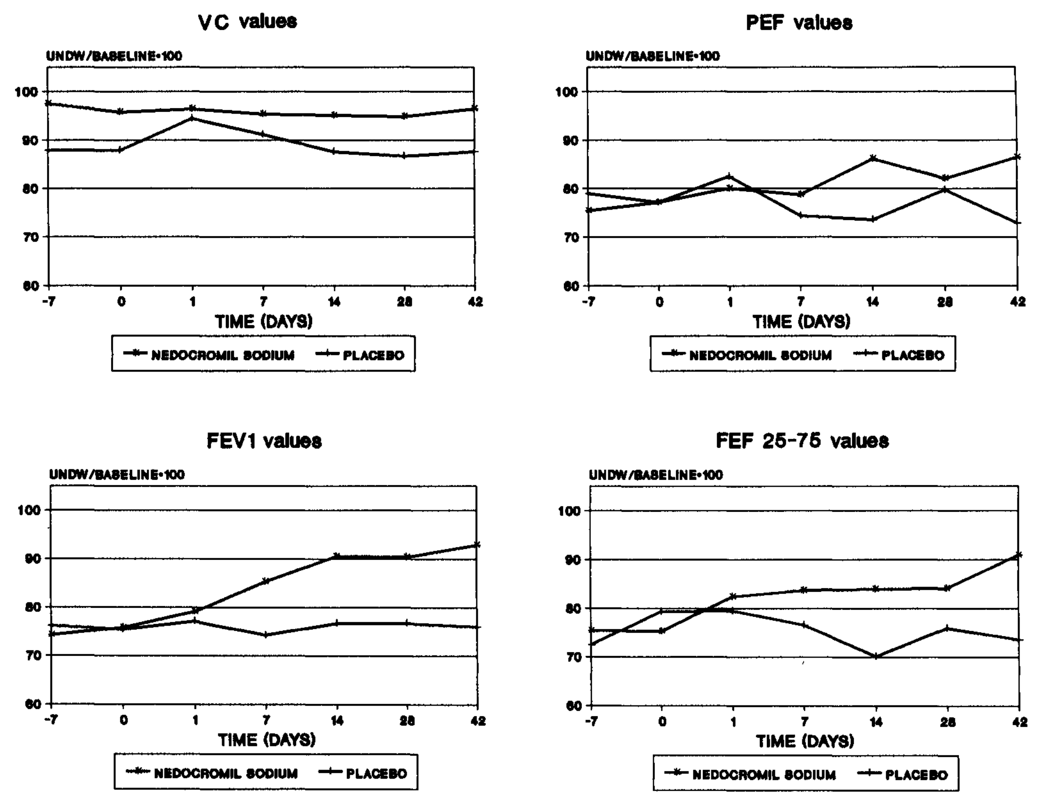

FIG. 1. Spirometric parameters at baseline in treated and placebo group (percentage of expected value): $p$ = N.S. at any time.

**Table 2.** Median ± SE and percentage of vital capacity (after UNDW/baseline) in groups N and P at different times

| Time | Group N | Group P | *p* |
|---|---|---|---|
| -7 | 92.5 ± 1.25 | 89.9 ± 3.04 | 0.12 |
| 0 | 90.7 ± 0.66 | 88.9 ± 2.97 | 0.14 |
| 1 | 96.4 ± 0.79 | 94.5 ± 2.05 | 0.02 |
| 7 | 95.4 ± 4.25 | 91.1 ± 1.62 | 0.12 |
| 14 | 95.1 ± 1.40 | 87.6 ± 2.38 | 0.02 |
| 28 | 94.8 ± 1.30 | 86.7 ± 3.42 | 0.04 |
| 42 | 96.4 ± 1.42 | 87.6 ± 1.89 | 0.01 |

**Table 3.** Median ± SE and percentage of FEV1 (after UNDW/baseline) in groups N and P at different times

| Time | Group N | Group P | *p* |
|---|---|---|---|
| -7 | 74.2 ± 0.94 | 76.1 ± 2.58 | 0.80 |
| 0 | 75.8 ± 0.83 | 75.4 ± 1.03 | 0.80 |
| 1 | 79.0 ± 1.02 | 77.1 ± 2.06 | 0.37 |
| 7 | 85.2 ± 1.39 | 74.2 ± 0.95 | 0.01 |
| 14 | 90.6 ± 1.04 | 76.7 ± 1.11 | 0.004 |
| 28 | 90.5 ± 2.73 | 76.7 ± 3.50 | 0.06 |
| 42 | 92.8 ± 0.97 | 76.0 ± 0.77 | 0.004 |

significant decrease in bronchoreactivity in group N (Table 4). FEF 25-75 changes after UNDW, which were initially comparable, showed a progressive decrease in bronchoreactivity in group N, and no change in group P (Table 5).

### Discussion

Although changes in bronchial reactivity have been reported in adult and young asthmatic patients, few attempts have been made to identify the cause of this behaviour. In allergic children, bronchial reactivity may increase during the season of exposure to the allergen.[14,15] Children affected by episodic asthma or by seasonal, non-atopic, recurrent asthma, whose hyper-reactivity was seasonal (i.e. it appeared in winter and disappeared in summer) were studied. In these cases, alteration in reactivity may reflect an inflammatory process induced, in winter, by such

**Table 4.** Median ± SE and percentage of PEF (after UNDW/baseline) in groups N and P at different times

| Time | Group N | Group P | *p* |
|---|---|---|---|
| -7 | 75.3 ± 2.52 | 78.9 ± 3.73 | 0.68 |
| 0 | 77.0 ± 1.95 | 77.1 ± 1.94 | 0.68 |
| 1 | 80.0 ± 4.20 | 82.5 ± 4.03 | 0.80 |
| 7 | 78.6 ± 2.65 | 74.3 ± 2.73 | 0.16 |
| 14 | 86.1 ± 2.09 | 73.5 ± 4.76 | 0.12 |
| 28 | 82.1 ± 2.37 | 79.7 ± 2.62 | 0.29 |
| 42 | 86.4 ± 2.13 | 72.9 ± 2.32 | 0.01 |

**Table 5.** Median ± SE and percentage of FEF 25-75 (after UNDW/baseline) in groups N and P at different times

| Time | Group N | Group P | *p* |
|---|---|---|---|
| -7 | 75.5 ± 7.21 | 72.4 ± 6.71 | 0.80 |
| 0 | 75.2 ± 4.98 | 79.3 ± 4.17 | 0.56 |
| 1 | 82.4 ± 3.68 | 79.4 ± 6.29 | 0.46 |
| 7 | 83.7 ± 1.29 | 76.6 ± 6.57 | 0.02 |
| 14 | 83.9 ± 2.39 | 70.1 ± 5.36 | 0.04 |
| 28 | 83.9 ± 2.10 | 75.9 ± 3.07 | 0.02 |
| 42 | 90.9 ± 4.24 | 73.5 ± 6.95 | 0.16 |

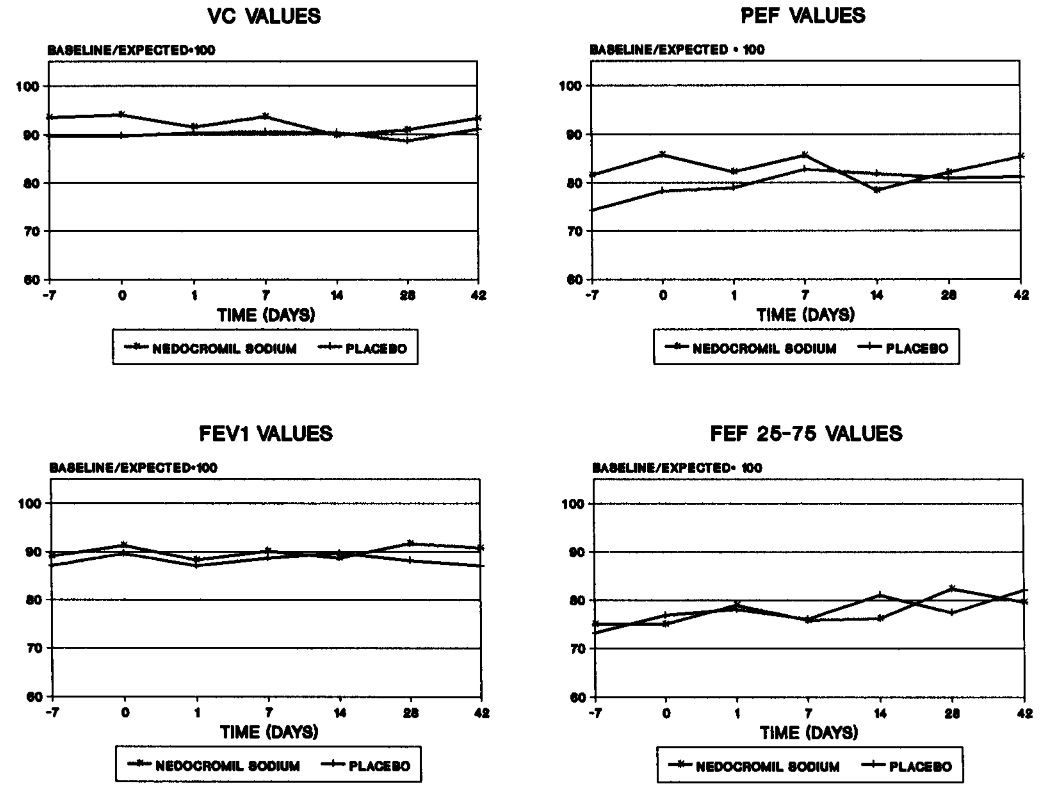

FIG. 2. Spirometric parameters after UNDW challenge in treated and placebo group (percentage of pre-challenge value): for significance, see Tables 2–5.

factors as cold, exposure to viral infection, and atmospheric pollutants. These patients, who were somewhat rare, were selected in order to test the efficacy of nedocromil sodium in extrinsic non-allergic asthma.

The results of this study indicate that nedocromil sodium was beneficial in these patients. From the second week of treatment, patients showed a better spirometric performance, and maintained good clinical conditions. Despite the small number of subjects and the relatively short study period, the data are statistically significant: this confirms the efficacy of nedocromil sodium as a prophylactic treatment.

No difference in functional parameters was found between the two groups in baseline conditions, but there was a progressive improvement in the values of the N group measured after UNDW. This is consistent with clinical data and recruitment criteria (i.e. children with episodic asthma, whose resting respiratory function was very close to normal); functional disorders only occurred after aspecific challenge. To our knowledge, our data are the first to show that nedocromil sodium affords protection against UNDW challenge in asthmatic children.

Our results are consistent with previous reports of preadministration of 2 and 4 mg of nedocromil sodium, 10–30 min before aspecific challenge prevents bronchospasm induced by UNDW.[17] However, we performed the test at least 6 h after administration of nedocromil sodium; therefore, the effect obtained cannot be attributed to an immediate inhibition, but rather to a reduction in the airway inflammation underlying the hyper-reactivity. Nedocromil sodium is known to exhibit a series of anti-inflammatory activities: it prevents the generation of neutrophil chemotactic peptides as IL-8,[23] inhibits human neutrophil chemotaxis,[24,25] decreases the bronchospastic response induced by $SO_2$ inhalation in animal models,[26] and prevents infiltration of neutrophils from bronchial mucosa of dogs exposed to ozone.[8]

Recent findings suggest that nedocromil sodium downregulates activation of cells involved in the late-phase asthmatic reaction, such as eosinophils,[27] mast-cells and T-cells.[28] Although it inhibits the development of mucosal inflammation, it was uncertain whether nedocromil sodium eliminates or reduces existing inflammatory changes.[29] Our data suggest that it can do this, as clinical trials have demonstrated its efficacy in diminishing the late phase asthmatic reaction. Therefore, the drug not only prevents the seasonal increase in aspecific bronchial reactivity as previously reported in patients naturally exposed to allergens,[15,16] but it is also useful in the treatment of non-allergic asthma.

In conclusion, nedocromil sodium, which may be used as prophylaxis in atopic asthmatic subjects with symptoms and bronchial hyper-reactivity upon natural exposure to allergens, may also be used to treat children suffering from episodic asthma, in whom airway inflammation increases bronchial reactivity.

### References

1. Boushey HA, Holtzman MJ, Sheller JR. Bronchial hyper-reactivity. *Am Rev Respir Dis* 1980; **121:** 389–394.
2. Townley RG, Ryo UY, Koloktin SM, Kang B. Bronchial sensitivity to methacholine in current and former asthmatic and allergic rhinitis and control patients. *J Allergy Clin Immunol* 1975; **56:** 429–434.
3. Juniper EF, Frith PA, Hargreave FE. Airway responsiveness to hystamine and methacholine: relation to minimal treatment to central symptoms of asthma. *Thorax* 1981; **36:** 575–579.
4. Cloutier MM, Loughlin GM. Chronic cough in children: a manifestation of airway hyper-reactivity. *Pediatrics* 1981; **67:** 6–8.
5. Barnes PJ. New concepts in the pathogenesis of bronchial hyper-responsiveness and asthma. *J Allergy Clin Immunol* 1989; **83:** 1013–1026.
6. Hargreave FE, Ramsdale EH, Kirby JG, O'Byrne PM. Asthma and the role of inflammation. *Eur J Respir Dis* 1986; **69:** 16–20.
7. Britton JR, Burney PGJ, Chinn S, Papacosta AO, Tattersfield AE. The relation between changes in airway reactivity and change in respiratory symptoms and medications in a community survey. *Am Rev Respir Dis* 1988: **138:** 530–534.
8. Di Stefano A, Chitano P, Saetta M. Nedocromil sodium inhibits airway mucosa neutrophilia and increased vascularity induced by ozone in dogs. *Eur Respir J* 1988; **1:** 182s–185s.
9. Slapke J, Vucelic B, Schutt C, Muller S. Der Einfluss klimaticher und metereologischer Faktoren auf die bronchiale Hyper-reaktivitat und den Verlauf von Asthma-bronchiale-Erkrankungen und ihre potentielle Bedeutung in der Asthmaprophylaxe: Hypotesen, metodische Ansatze und erste Erkenntnisse. *Z Erkr Atmungsorgane* 1989; **173:** 116–121.
10. Richards W. Effects of air pollution on asthma. *Ann Allergy* 1990; **65:** 345–347.
11. Spinaci S, Arossa W, Bugiani M, Natale P, Bucca C, de Candussio G. The effects of air pollution on the respiratory health of children: a cross-sectional study. *Pediatr Pulmonol* 1985; **1:** 262–266.
12. Braback L, Kalvesten L. Urban living as a risk factor for atopic sensitization in Swedish schoolchildren. *Pediatr Allergy Immunol* 1991: **2:** 14–19.
13. Henry R, Abramson R, Adler J, Wlodarcyzk J, Hensley M. Asthma in the vicinity of power stations. I. A prevalence study. *Pediatr Pulmonol* 1991; **11:** 127–133.
14. Boner AL, Piacentini GL, Bonizzato L, Dattoli V, Sette L. Effect of inhaled beclomethasone dipropionate on bronchial hyper-reactivity in asthmatic children during maximal allergen exposure. *Pediatr Pulmonol* 1991; **10:** 2–8.
15. Dorward AJ, Roberts JA, Thomson NC. Effect of nedocromil sodium on histamine airway responsiveness in grass pollen sensitive asthmatics during the pollen season. *Clin Allergy* 1986; **16:** 309–314.
16. Altounyan REC, Cole M, Lee TB. Effect of nedocromil sodium on changes in bronchial hyper-reactivity in non-asthmatic, atopic, rhinitic subjects during the grass pollen season. *Eur J Respir Dis* 1986; **69:** 271s–273s.
17. Robuschi M, Vaghi A, Simone P, Bianco S. Prevention of fog-induced bronchospasm by nedocromil sodium. *Clin Allergy* 1987; **17:** 69–74.
18. Altounyan REC, Cole M, Lee TB. Inhibition of sulfur dioxide-induced bronchoconstriction by nedocromil sodium and sodium cromoglycate in non-asthmatic atopic subjects. *Eur J Respir Dis* 1986; **69:** 274s–277s.
19. Crimi E, Brusasco V, Brancatisano M. Effect of nedocromil sodium on adenosine- and methacoline-induced bronchospasm in asthma. *Clin Allergy* 1987; **17:** 135–139.
20. Crimi E, Brusasco V, Crimi P. Effect of nedocromil sodium on the late asthmatic reaction to bronchial antigen challenge. *J Allergy Clin Immunol* 1989; **83:** 303–312.
21. Allegra L, Bianco S. Non-specific broncho-reactivity obtained with an ultrasonic aerosol of distilled water. *Eur J Respir Dis* 1980; **61:** 41–49.
22. Zapletal A, Samanek M, Paul T. *Lung Function in Children and Adolescents*. Basel: Karger, 1987.
23. Vittori E, Sciacca F, Colotta F, Mantovani A, Mattoli S. Protective effect of nedocromil sodium on the Interleukin 1-induced production of Interleukin 8 in human bronchial epithelial cells. *J Allergy Clin Immunol* 1992; **90:** 76–84.
24. Moqbel R, Walsh GM, Kay AB. Inhibition of human granulocyte activation by nedocromil sodium. *Eur J Respir Dis* 1986; **69:** 227s–280s.
25. Chitano P, Maestrelli P, Mapp CE, Rainey K, Fabbri LM, Allegra L. Nedocromil sodium and disodium cromoglycate inhibit human neutrophil chemotaxis *in vitro*. *Am Rev Respir Dis* 1989; **139** (Suppl): A370.
26. Jackson DM, Eady RP. Acute transient $SO_2$-induced airway hyper-reactivity: effect of nedocromil sodium. *J Appl Physiol* 1988; **65:** 1119–1123.
27. Sedgwick JB, Bjornsdottir U, Geiger KM, Busse WW. Inhibition of eosinophil density change and leukotriene C4 generation by nedocromil sodium. *J Allergy Clin Immunol* 1992; **90:** 202–209.
28. Mekori YA, Baram D, Goldberg A, Hershkoviz R, Reshef T, Sredni D. Nedocromil sodium inhibits T-cell function *in vitro* and *in vivo*. *J Allergy Clin Immunol* 1993; **91:** 817–824.
29. Dahl R, Haahtela T. Prophylactic pharmacological treatment of asthma. *Allergy* 1992; **47:** 588–593.
